# Morphology and novel classification of proximal humeral fractures

**DOI:** 10.3389/fbioe.2024.1366089

**Published:** 2024-07-19

**Authors:** Jichao Liu, Ziyan Zhang, Jie Ding, Jian Zhang, Qian Sheng, Chengdong Piao

**Affiliations:** ^1^ Department of Orthopedics, The Second Hospital of Jilin University, Changchun, Jilin Province, China; ^2^ Department of Stomatology, The Affiliated Hospital of Changchun University of Traditional Chinese Medicine, Changchun, Jilin Province, China; ^3^ Radiology Department, The Second Hospital of Jilin University, Changchun, Jilin Province, China; ^4^ Medical Insurance Office, The Second Hospital of Jilin University, Changchun, Jilin Province, China

**Keywords:** proximal humeral fracture, fracture mapping, classification, pathomorphology, reliability, three-dimensional CT

## Abstract

**Background:** The morphology of proximal humeral fractures (PHFs) is complex, and the fixation and selection of implants need to be guided by the fracture type and classification, which requires an accurate understanding of the fracture line. This study had three purposes. 1) Define and analyze the fracture lines and morphological features of all types PHFs by three-dimensional (3D) mapping technology. 2) Determine the osteotomy position of the biomechanical model of the PHFs according to the fracture heat map. 3) Based on the analysis of the pathological morphology and distribution of a large number of consecutive cases of PHFs, propose a novel classification of PHFs.

**Methods:** We retrospectively collected 220 cases of PHFs and generated a 3D fracture map and heat map based on computed tomography (CT) imaging. Through analysis of the fracture morphology of the 220 PHFs, a novel classification was proposed. The primary criterion for staging was the continuity between the humeral head and the greater tuberosity and lesser tuberosity, and the secondary criterion was the relationship between the humeral head segment and the humeral shaft.

**Results:** The fracture line was primarily found around the metaphyseal zone of region of the surgical neck, with the most extensive distribution being below the larger tuberosity and on the posterior medial side of the epiphysis. We suggest that the osteotomy gap should be immediately (approximately 5–10 mm) below the lower edge of the articular surface. The most common type of fracture was type I3 (33 cases, 15.0%), followed by type IV3 fracture (23 cases, 10.4%), and type III2 fracture (22 cases, 10.0%). Interobserver and intraobserver reliability analysis for the fracture classification revealed a k value (95% confidence interval) of 0.639 (0.57–0.71) and 0.841, *P* < 0.01, respectively.

**Conclusion:** In this study, the fracture line and morphological characteristics of PHFs were clarified in detail by 3D mapping technique. In addition, a new classification method was proposed by analysis of the morphological characteristics of 220 PHFs, A two-part fracture model for PHFs is also proposed.

## Introduction

Proximal humeral fractures (PHFs) are common, especially in the elderly population, and represent 4% of all adult fractures (Badman and Mighell, 2008). The incidence of complex fracture types tends to be positively correlated with increasing age, predominantly because older people have less bone density and mobility (Huang, 2019). PHFs have attracted increasing attention as the effects of an aging society become more pronounced (Sun et al., 2020). Severe osteoporosis and decreased bone mineral density in the elderly means the treatment of PHFs is challenging (Tamimi et al., 2015; Mease et al., 2021). Detailed understanding of the morphology and distribution characteristics of fracture lines in PHFs and classification of fractures can effectively guide treatment and determine the prognosis of fractures (Yimam et al., 2022).

Fracture mapping, initially proposed by Armitage et al. (2009), is a method to visually display the fracture morphology by superimposing fracture lines of multiple fracture models onto a normal model through 3D CT reconstruction (Guo et al., 2021a; Guo et al., 2021b). The fracture map provides a visual representation of the position, direction, and distribution of the fracture line (Ding et al., 2022). This visual representation allows doctors to better understand the distribution and shape of the fracture line and can also support the development of a more scientific classification for fractures (Yao et al., 2022). The existing fracture mapping studies of PHFs are mostly limited to complex fractures (three-and four-part PHFs), while fracture map studies covering all fracture types are rare (Hasan et al., 2017; Ju et al., 2022; Mys et al., 2023). Ren et al. (2023) recently retrospectively collected 312 cases of proximal humeral fractures, analyzed the patterns of PHFs by 3D mapping, and found that these different fracture patterns may be closely related to different clinical prognoses. However, they did not make a further study on the morphology of fracture lines in all patients.

The Neer classification and AO classification for PHFs are both widely recognized (Neer, 1970; Caviglia et al., 2002). However, there is insufficient interobserver agreement for these two classifications (Papakonstantinou et al., 2016). Furthermore, the intricacy of AO categorization means this system has limited clinical use and no clear benefit in the examination of fractures (Hertel et al., 2004; Resch et al., 2016). Other classification systems for PHFs are predominantly based on five basic fracture surfaces among the four anatomical structures—humeral head, greater tuberosity, lesser tuberosity, and humeral shaft—and thus divide PHFs into a range of basic fracture types (Hertel et al., 2004; Sukthankar et al., 2013). Nevertheless, there is currently no classification system that can explain the biomechanical mechanism of fracture and describe the displacement of humeral head, greater tuberosity, and lesser tuberosity (Resch et al., 2016). In addition, in biomechanical studies related to PHFs, there is no consensus on the osteotomy position of a two-part fracture model of PHFs (Schumer et al., 2010; Lammi et al., 2014; Zhang et al., 2016; Acklin et al., 2018).

This study was conducted to further explore the morphology of PHFs and had three aims. 1) Define and analyze the fracture lines and morphological features of all types PHFs by 3D mapping technology. 2) Determine the osteotomy position of the biomechanical model of the PHFs according to the fracture heat map. 3) Based on the analysis of the pathological morphology and distribution of a large number of consecutive cases of PHFs, propose a new classification that can explain the biomechanical mechanism (including flexion and extension) of fracture and describe the displacement of humeral head, greater tuberosity, and lesser tuberosity to help doctors evaluate the injury.

## Materials and methods

### Subjects

A retrospective search of PHFs from January 2018 to November 2022 was conducted at The Second Hospital of Jilin University. The study was approved by the hospital ethics committee.

A total of 256 patients with PHFs were screened during this period. The inclusion criteria are as follows: 1) complete demographic data; 2) over 18 years old; 3) closed fracture; 4) preoperative shoulder CT scan in our hospital, and reliable imaging data are sufficient for quantitative 3D CT modeling. The exclusion criteria were as follows: 1) pathological fracture (except osteoporosis); 2) open fracture; 3) multiple injuries; 4) poor quality of CT scan data (slices thickness larger than 3 mm, incomplete scan images); 5) previous shoulder surgery, rheumatism, congenital or acquired shoulder deformity, primary glenohumeral osteoarthritis. Twelve patients were excluded because they were under 18 years old, ten patients were excluded due to lack of preoperative CT images, six patients were excluded for poor CT image quality, five patients were excluded for open fractures, two patients were excluded for previous shoulder injuries, and one patient was excluded for rheumatoid arthritis.

### Fracture mapping

**FIGURE 1:**
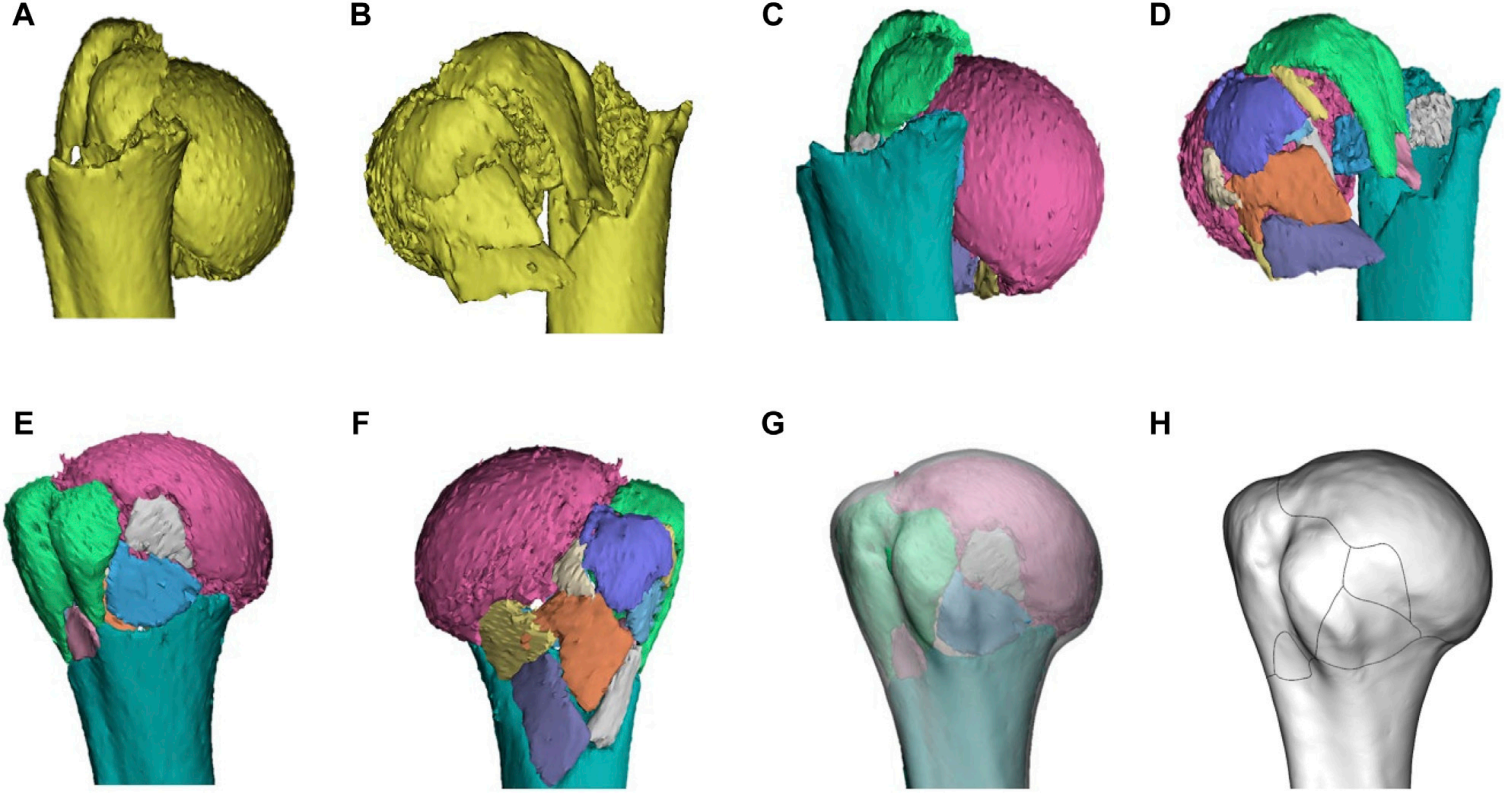
Representative images of steps in the method used for three-dimensional mapping of proximal humeral fractures. In this example of a proximal humeral fracture, each fragment was reconstructed **(A, B)**, segmented **(C, D)**, and virtually reduced **(E, F)**. The fracture is then mirrored (if left) and matched to the model **(G)** of 1/3 of the proximal humerus. The osseous contours of the proximal humerus, especially greater tuberosity, lesser tuberosity, intertubercular groove, and anatomical neck, are referenced for alignment and standardization. The contour of every fracture fragment was marked with smooth curves to delineate the fracture lines **(H)**.

The preoperative CT images of all patients included in this study were derived. A healthy adult male model of 3D CT reconstruction of the right humerus (26 years old, with no history of shoulder trauma) was selected as the standard proximal humeral model. The process of fracture mapping is shown in Figure 1.

(1) The CT scan files for the patients were exported in Digital Imaging and Communication in Medicine (DICOM) format and then reconstructed in 3D using the Mimics 21.0 system (Materialise, Leuven, Belgium). In mimics, use the “threshold segmentation” tool to reconstruct the Mask of fracture model, and then use the “edit,” “split mask” and “region grow” tools to separate the fracture fragments. Finally, use “calculate part” to output the model in STL format.
(2) The reconstructed model was outputted to 3-Matic 13.0 software (Materialise, Leuven, Belgium) in STL format. Use the “Interactive Translate” and “Interactive Rotate” tools to move and rotate the fragments to reduce the fracture. All left PHF patients use “Mirror” to flip, and model size was standardized use “Scale” tool to maximize the matching of the normal proximal humerus model. A series of anatomical markers were established for the standard proximal humeral model, including the humeral head, greater tuberosity, lesser tuberosity, internodular sulcus, and anatomical neck of the humerus. Adjust the transparency of the 3D standard model to “High,” then use the “Interactive Translate” and “Interactive Rotate” tools to overlay the normalized fracture model on the standard model and align the anatomical marks. Finally, the “Create Curve” tool in 3-Matic software is used to draw the fracture line. If necessary, a close curve can be applied. Each fracture line was renamed, and patient information and number were recorded for subsequent analyses.
(3) Input the standard model and fracture line into E-3D (Central South University, Changsha, China) in STL and txt format respectively, and use the “Fracture line statistical analysis” tool in the “Analysis and processing” section to generate a heat map based on the fracture line.

### Evaluation and classification of fracture

The threshold of 20° was determined for coronal and sagittal head fragment malposition (Solberg et al., 2009; Tauber et al., 2015).

**FIGURE 2:**
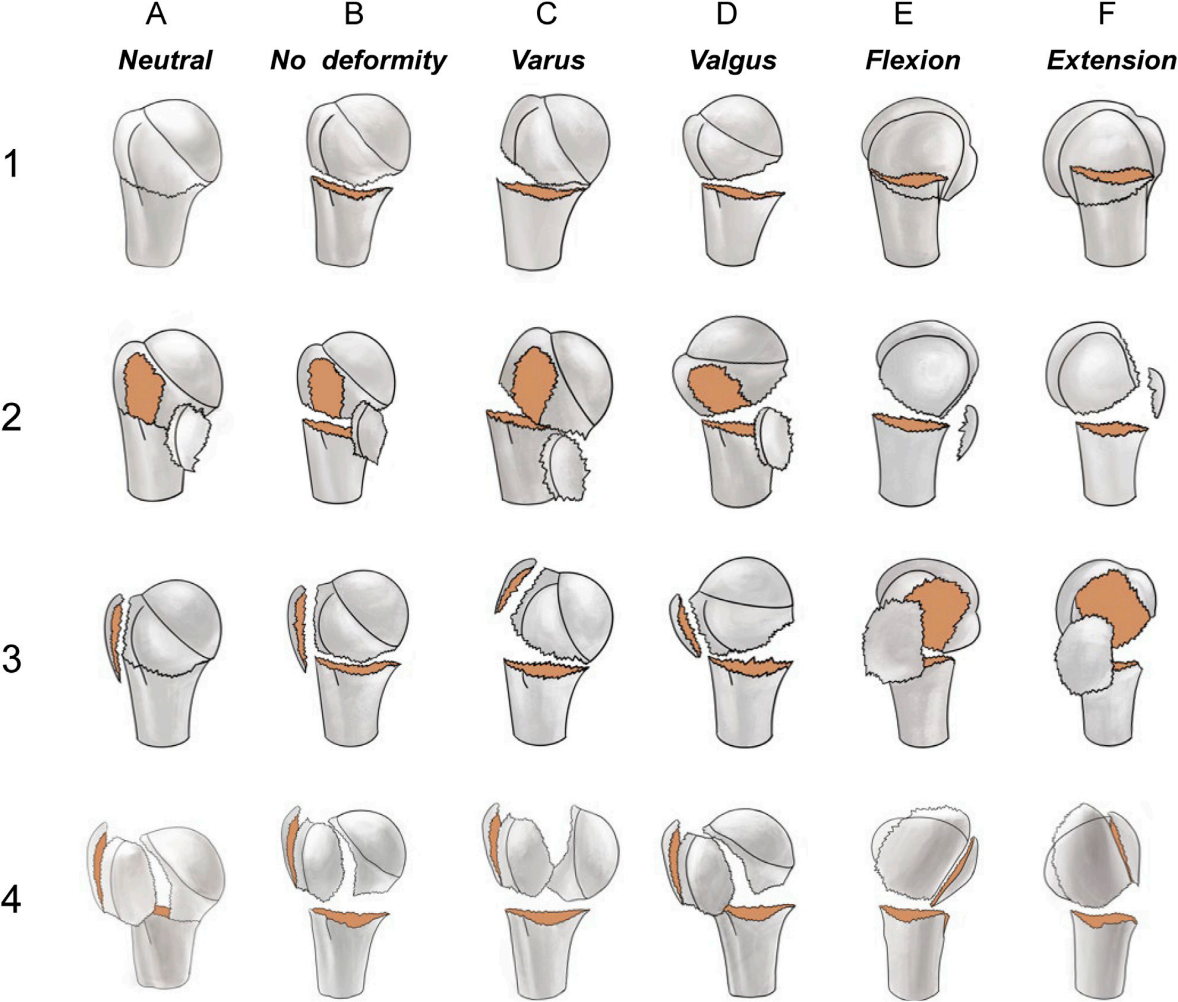
The primary categorization criterion (1, 2, 3, and 4) was determined as the continuity of the larger and lesser tuberosities with the humeral head. Type 1: the humeral head is continuous with both the greater and lesser tuberosities; Type 2: the humeral head is continuous with the greater tuberosity only; Type 3: the humeral head is continuous with the lesser tuberosity only; Type 4: the humeral head is not connected with the greater and lesser tuberosities. The secondary criterion of classification is the position relationship between humeral head and shaft segment, and is divided into six types A–F). Type A is a surgical neck with no fracture or a fracture that does not meet the criteria for displacement; type B is a surgical neck fracture but the humeral head segment is in a neutral position; type C is a varus malposition; type D a valgus malposition; type E is an anterior tilt; and type F is a posterior tilt.

**FIGURE 3:**
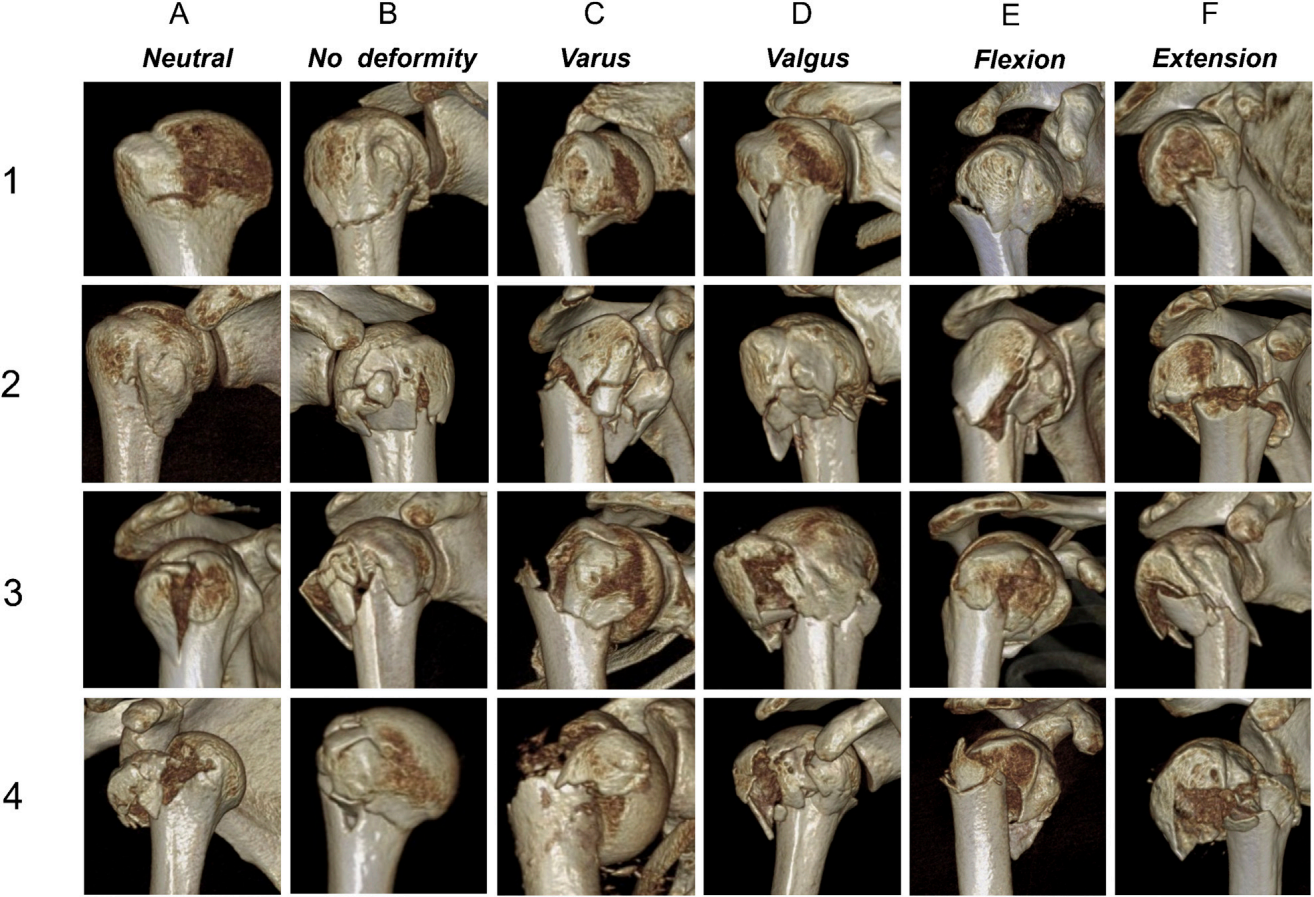
Three-dimensional computed tomography examples for each fracture. (1–4) The main classification criteria; (A–F) The secondary classification criteria.

Figure 2 shows the schematic diagram of the novel classification. Examples of 3D CT for each fracture are shown in Figure 3. The primary categorization criterion was determined as the continuity of the greater and lesser tuberosities with the humeral head and comprised four types (1–4). Type 1: the humeral head is continuous with the greater and lesser tuberosities; type 2: the humeral head is continuous with the greater tuberosity only; type 3: the humeral head is connected with the lesser tuberosity only; and type 4: the humeral head is not connected with the greater and lesser tuberosities. The secondary criterion of classification was determined as the position of the relationship between head and shaft segment, and was divided into six types (A–F). Type A is a surgical neck with no fracture or a fracture that does not meet the criteria for displacement; type B is a surgical neck fracture but the humeral head segment is in a neutral position; type C is varus deformity; type D is valgus deformity; type E is flexion deformity; and type F is extension deformity.

Three observers assessed fracture morphology based on a structured questionnaire (Table 1), which was a binary description system that allowed the fracture morphology of different cases to be described with yes/no responses. Based on the recorded responses, the evaluations of the three observers were combined to determine the pathological pattern of the fracture and an interobserver agreement analysis was performed. If the three assessments for a fracture did not concur, the fracture was reevaluated and discussed. If the evaluators reached an agreement, the initial evaluation was adjusted; otherwise, the evaluation was left unaltered. The results were then aggregated to analyze the interobserver agreement of the three observers. In addition, to assess intraobserver reliability, one observer repeated the procedure in a randomized manner 4 weeks after the first assessment.

**TABLE 1:** Structured questionnaire for fracture morphological analysis.

	Yes	No
1. Questions relating to greater tuberosity		
Is there a fracture between the head and the greater tuberosity?	□	□
If yes, is the displacement from the head greater than 10 mm?	□	□
If yes, is the angulation greater than 45°?	□	□
2. Questions relating to lesser tuberosity		
Is there a fracture between the head and the lesser tubercle?	□	□
If yes, is the displacement from the head greater than 10 mm?	□	□
If yes, is the angulation greater than 45°?	□	□
3. Questions relating to humeral shaft		
Is there a fracture between the head (Humeral head and the part of the humerus to which it is attached) and the humeral shaft?	□	□
If yes, is the displacement between posteromedial edge of the head and posteromedial shaft fracture line >10 mm	□	□
4. Displacement of the head in the sagittal plane		
Is the head angled in the sagittal plane?	□	□
Is the angulation >20°?	□	□
Is it anteriorly inclined?	□	□
Is it posteriorly inclined?	□	□
5. Displacement of the head in the coronal plane?		
Is the head angled in the coronal plane?	□	□
Is the angle >20°?	□	□
Is it inversion?	□	□
Is there an ectropion?	□	□

## Statistics

Cohen or Fleiss k statistics were used to calculate the intraobserver and interobserver reliabilities, including 95% confidence intervals, and Landis and Koch criteria were used to judge the level of the agreement (0.00–0.20 slight agreement, 0.21–0.40 fair, 0.41–0.60 moderate, 0.61–0.80 substantial, 0.81–1.00 almost perfect). Data analysis was performed using SPSS software version 26 (IBM Corp, Armonk, NY, United States).

## Results

The study included 220 consecutive patients—146 females and 74 males—with PHF. The mean age of all patients was 58.3 ± 16.7 years, while the mean age for males and females was 50.8 ± 15.1 years and 62.0 ± 16.0 years, respectively (*P* < 0.05). There were 108 left shoulders and 112 right shoulders. Demographic characteristics of the patients are shown in Table 2.

**TABLE 2:** Patient demographic characteristics.

	Characteristics	
Patients (n = 220)	Sex ratio (Female/Male)	146/74 (1:0.51)
	Age (±SD, years)	58.3 ± 16.7
	Age (±SD, years; Female/Male)	62.0 ± 16.2/50.8 ± 15.1
Shoulder	Left	112 (50.9%)
	Right	108 (49.1%)

**FIGURE 4:**
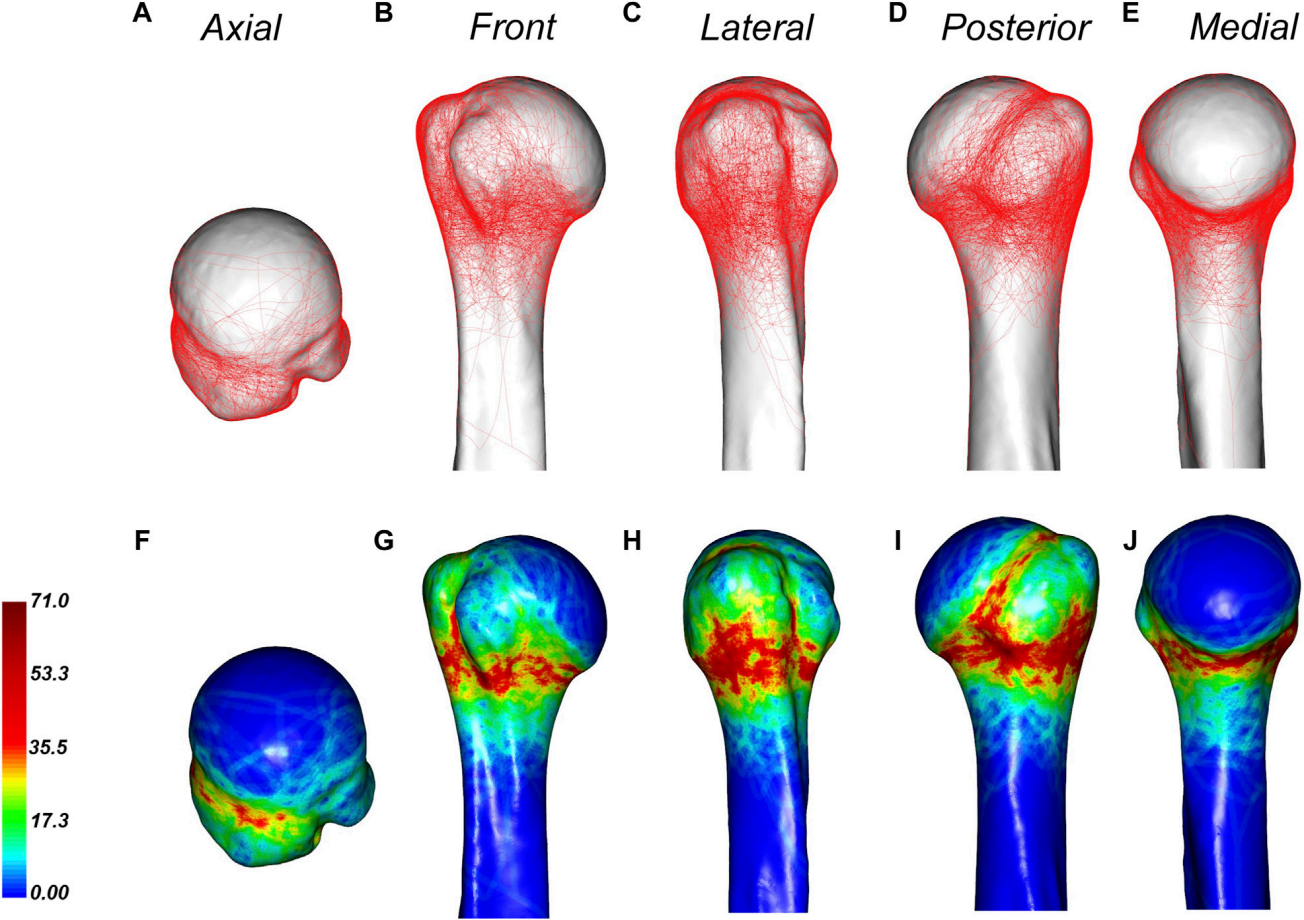
General maps of the hot zones of 3D fracture lines of all PHFs. **(A–E)**, representative views of the proximal humerus. **(F–J)**, 3D heat mapping superimposed with all proximal humeral fracture lines (*n* = 220), including the axial, front, lateral, posterior, and medial views. Red represents a higher frequency of fracture line density.

Figure 4 displays the 3D fracture map and heat map of PHFs. The fracture line was primarily found around the metaphyseal region of the surgical neck, with the most extensive distribution being below the greater tuberosity and on the posterior medial side of the epiphysis. The fracture line below the greater tuberosity exhibited a cross-shaped distribution; a vertical fracture line was more common in the greater tuberosity, while a horizontal fracture line was more common in the epiphysis. A dense area of fracture lines in the posterior medial aspect of the diaphysis was distributed along the inferior border of the greater tuberosity. Greater tuberosity fractures were more common than lesser tuberosity fractures. Although fracture lines rarely involved the lesser tuberosity, there were dense areas at the junction of the lesser tuberosity and the epiphysis. Most fracture lines reach the lower part of the bicipital groove, and the fracture lines rarely pass through the bicipital groove. In addition, the fracture line rarely encroached on the joint surface.

**FIGURE 5:**
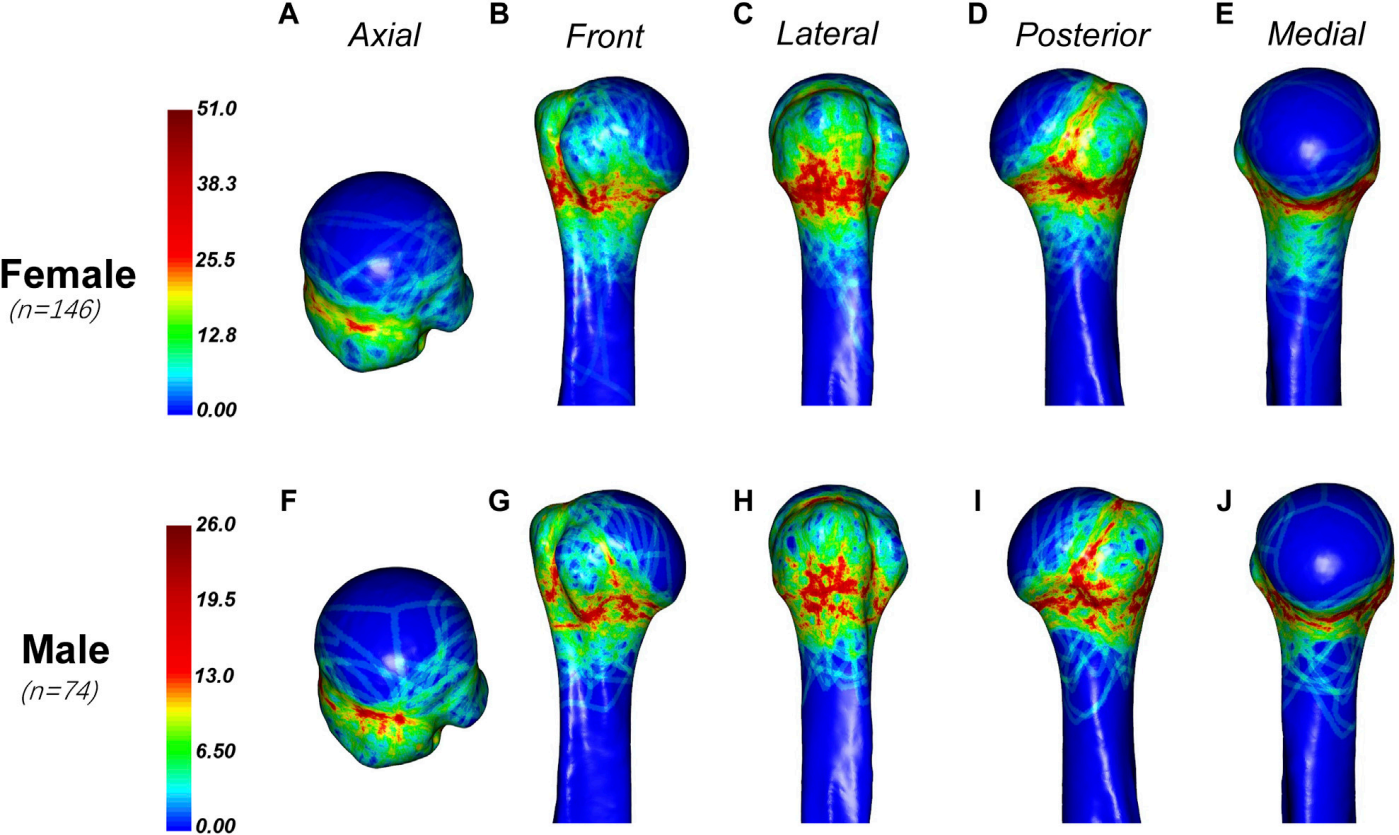
General maps of the hot zones of PHFs in male and female.Fracture lines in female patients **(A–E)** are more likely to involve the surgical neck, while in male patients **(F–J)**, they are more proximal in the dense area of the greater tuberosity. Male patients also tend to have fracture lines involving the anatomical neck, with a higher density of fracture lines in the upper, anterior, and posterior parts of the anatomical neck.

The distribution of fracture lines in male and female patients was slightly different (Figure 5). The fracture lines of male patients are more likely to involve the anatomical neck, and there are more dense fracture lines in the upper, anterior and posterior parts of the anatomical neck, while the fracture lines of female patients are more likely to involve the surgical neck. And, male patients are more proximal than female patients in the dense area of the fracture line of the greater tuberosity.

**FIGURE 6:**
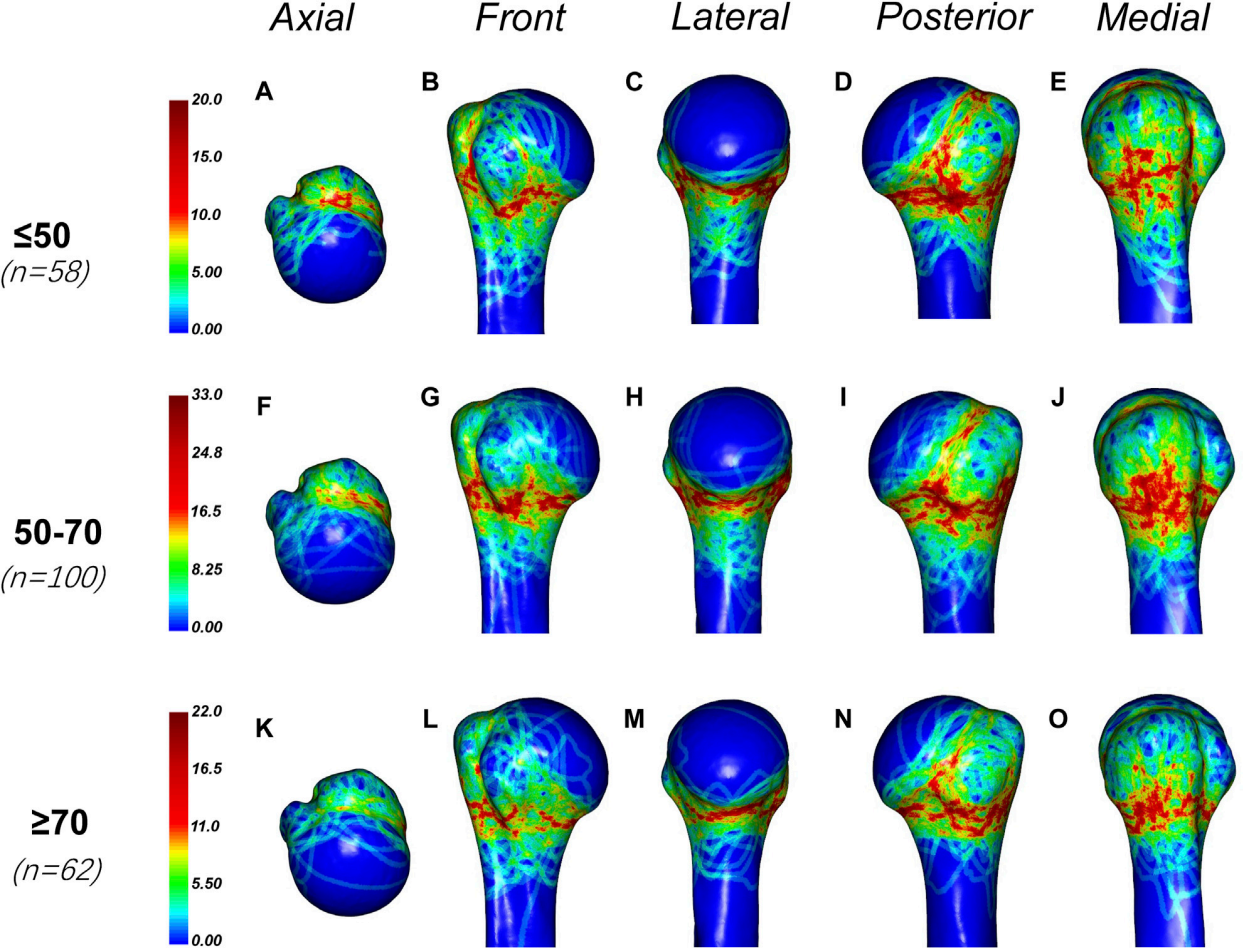
General maps of the hot zones of PHFs in different age groups. **(A–E)** Fracture heat maps for patients aged 50 years or younger; **(F–J)** Fracture heat maps for patients aged 50–70 years; **(K–O)** Fracture heat maps for patients aged 70 years or older. The fracture lines in each group were densely distributed in the epiphyseal region. However, fractures were more likely to occur the anatomical neck and bicipital groove in the younger group (less than or equal to 50  years old and 50–70 years old) than in the older group (greater than or equal to 70 years old). The fracture lines of patients over 70 years old were mainly distributed in the epiphysis.

In this study, we also found that the age of the patients affected the distribution of fracture lines. In this study, the patients were divided into three groups (less than or equal to 50 years old, 50–70 years old and greater than or equal to 70 years old), and the fracture heat maps of these three age groups were drawn respectively (Figure 6). We found that the fracture lines in each group were densely distributed in the epiphyseal region. However, fractures were more likely to occur the anatomical neck and bicipital groove in the younger group (less than or equal to 50 years old and 50–70 years old) than in the older group (greater than or equal to 70 years old). The fracture lines of patients over 70 years old were mainly distributed in the epiphysis.

**FIGURE 7:**
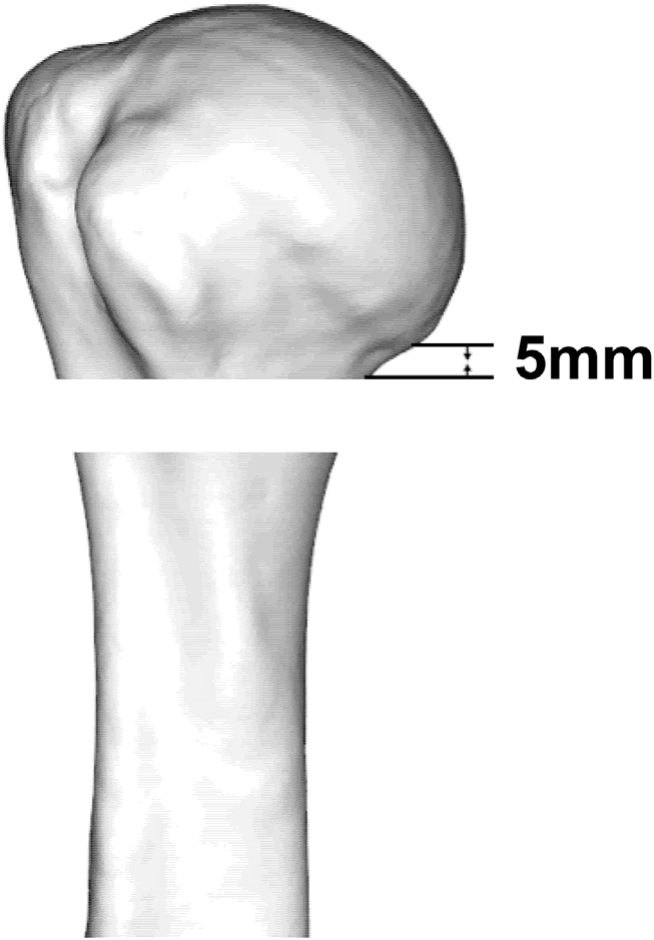
Osteotomy position of a two-part fracture model of the proximal humerus.

According to the fracture heat map, we suggest that the osteotomy gap should be immediately below the lower edge of the articular surface by approximately 5–10 mm, which can cover more fracture lines (Figure 7).

**FIGURE 8:**
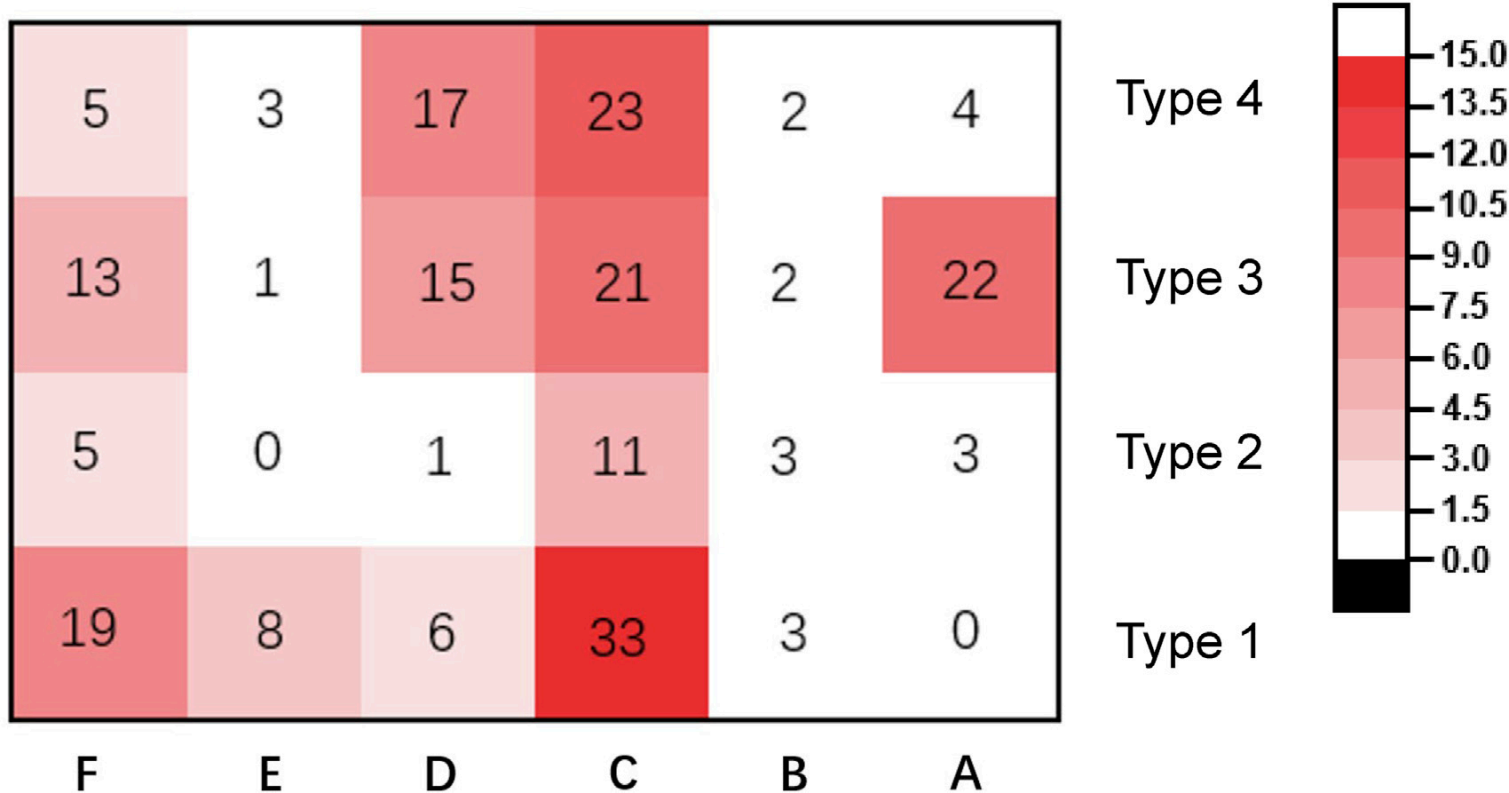
Frequency and number of different types of fracture in the patients included in the study.

Based on the analysis of fracture morphology, a novel classification of PHFs was proposed (Figure 2). The most common type of fracture was type 1C (33 cases, 15.0%), followed by type 4C (23 cases, 10.4%) and type 3B (22 cases, 10.0%). The frequency and number of each type of fracture are shown in Figure 8. Table 3 presents the results of the interobserver reliability analysis of the evaluations of the three independent observers. Interobserver and intraobserver reliability analysis for the fracture classification revealed a k value (95% confidence interval) of 0.639 (0.57–0.71) and 0.841, *P* < 0.01, respectively.

**TABLE 3:** Pathomorphologic findings and respective interobserver reliability.

Variable	%	k	95% CI
1. Questions relating to greater tuberosity			
Yes	0.49	0.704	0.628–0.780
No	0.31		
No agreement	0.20		
2. Questions relating to lesser tuberosity			
Yes	0.29	0.804	0.727–0.880
No	0.58		
No agreement	0.13		
3. Questions relating to humeral shaft			
Yes	0.80	0.859	0.783–0.935
No	0.15		
No agreement	0.06		
4. Displacement of the head in the sagittal plane			
Varus	0.34	0.843	0.787–0.899
Valgus	0.15		
Neutral (<20° angulation)	0.37		
No agreement	0.14		
5. Displacement of the head in the coronal plane			
Posterior tilt	0.19	0.836	0.778–0.894
Anterior tilt	0.03		
Neutral (<20° angulation)	0.68		
No agreement	0.10		

## Discussion

In this study, 3D fracture mapping was used to analyze 220 PHFs. The 3D mapping method can more precisely define fracture morphology compared with the 2D mapping technique (Dugarte et al., 2018). Using 3D mapping technology, we generated fracture maps and heat maps that visually showed the morphological characteristics of the fracture. In addition, the osteotomy position for the biomechanical model of PHFs was determined. Finally, through analysis of the morphology of 220 PHFs, we proposed a novel classification: combining the number of fracture fragments with the angle of head fragments relative to the shaft. This classification system reflects not only the degree of comminution of the fracture, but also the mechanical mechanisms of injury and degree of periosteal damage.

The average age of patients in this study was 58.3 ± 16.7 years (male, 50.8 ± 15.1 years; female, 62.0 ± 16 years), and a large proportion of the patients were female (146 patients, 66.4%), which may be because the bone mass of females decreases more significantly with age (Guggenbuhl et al., 2005; Yamada et al., 2007; Barvencik et al., 2010; Alidousti et al., 2017).

The existing fracture mapping studies of PHFs are mostly limited to complex fractures (three-and four-part PHFs). There are some similarities between their findings and ours. Hasan et al. (2017) previously investigated the morphology of 48 cases of complex PHFs. Although they used 2-D fracture line mapping, they confirmed good inter-rater reliability. They found that the fractures predominated at the surgical neck, fractures frequently split greater tuberosity, and fractures were less likely to involve the attachment points of the rotator cuff muscles, which was similar to our study (Hasan et al., 2017). Mys et al. (2023) recently published a study on fracture morphology, which explained the complex morphology of PHFs by mapping 50 cases of complex PHFs. Most of the findings were in line with our results, with a high frequency around the surgical neck and articular fractures were infrequent (Mys et al., 2023). However, despite many similarities with the research of Hasan et al. in fracture morphology, the incidence of fracture in the intramuscular groove and articular surfaces was significantly different. Hasan et al. (2017) found a high incidence of intraarticular fractures and that fractures were less likely to involve the intertubercular groove. In our study, fractures rarely involved the articular surface and often involve the inferior aspect of the bicipital groove. This may be related to the fact that the higher density of the articular surfaces was less susceptible to fracture, and the fractures in the patients included in the study by Hasan et al. may have been the result of stronger trauma (Tingart et al., 2003). In addition, Mys et al. (2023) found that the incidence of fracture in the anatomical neck was lower, while in our study, the incidence of fractures was higher in the lower, posterior, and upper parts of the anatomical neck. Thus, the fracture morphology of complex PHFs was slightly different from that of all types of PHFs (including simple fractures). These differences may be due to differences in the inclusion of patients. Moreover, the current studies on fracture maps of PHFs have included fewer patients. A recent 3D fracture morphological study by Ju et al. (2022) reported the fracture line through the intertubercular groove. However, their study focused only on fractures around the greater tuberosity. Ren et al. (2023) recently analyzed the patterns of PHFs using 3D mapping and found that these different fracture patterns may be closely related to different clinical prognoses. However, they only drew fracture maps of six fracture patterns, and did not take a further study of the shape and distribution of fracture lines in all patients.

The fracture morphology is closely related to the thickness of the cortical bone and the bone density. The thinner the cortical bone and the lower the bone density, the greater the probability of fracture. Epiphysis is the transition point from cancellous bone to dense bone, and the bone cortex is thinner and prone to fracture, so epiphysis was a dense area of fracture line. Another dense area of the fracture is below the greater tuberosity, which is the result of more serious bone loss with age. The bone cortex of the large nodule is thinner and the bone mineral density is lower, especially in women, so this area is more prone to osteoporotic fractures. Hepp et al. (2003) found the lowest bone strength in the greater tuberosity by indentation measurements on 24 cadaveric humeri. Similar conclusions were reached by Barvencik et al. (2010) who studied the bone density of the proximal humerus in 60 cadavers using X-rays and reported that the most significant decrease in bone density with age was in the greater tuberosity. Wang et al. (2018) conducted clinical CT scans of the proximal humerus of 103 healthy volunteers and found that there is usually a thinner humeral cortex in the posterior wall and the distal part of the lateral wall, which results from underloading of these areas during daily shoulder movements owing to the lack of muscle attachment. Tingart et al. (2003) used peripheral quantitative computed tomography (pQCT) to measure bone density in 20 human cadaver humeri and found that bone density was almost 30% higher in the lesser tuberosity compared with that in the greater tuberosity. In addition, the fracture line rarely involved the insertion of the rotator cuff muscles, suggesting that the fracture was related to the rotator cuff tendon (Hasan et al., 2017).

**FIGURE 9:**
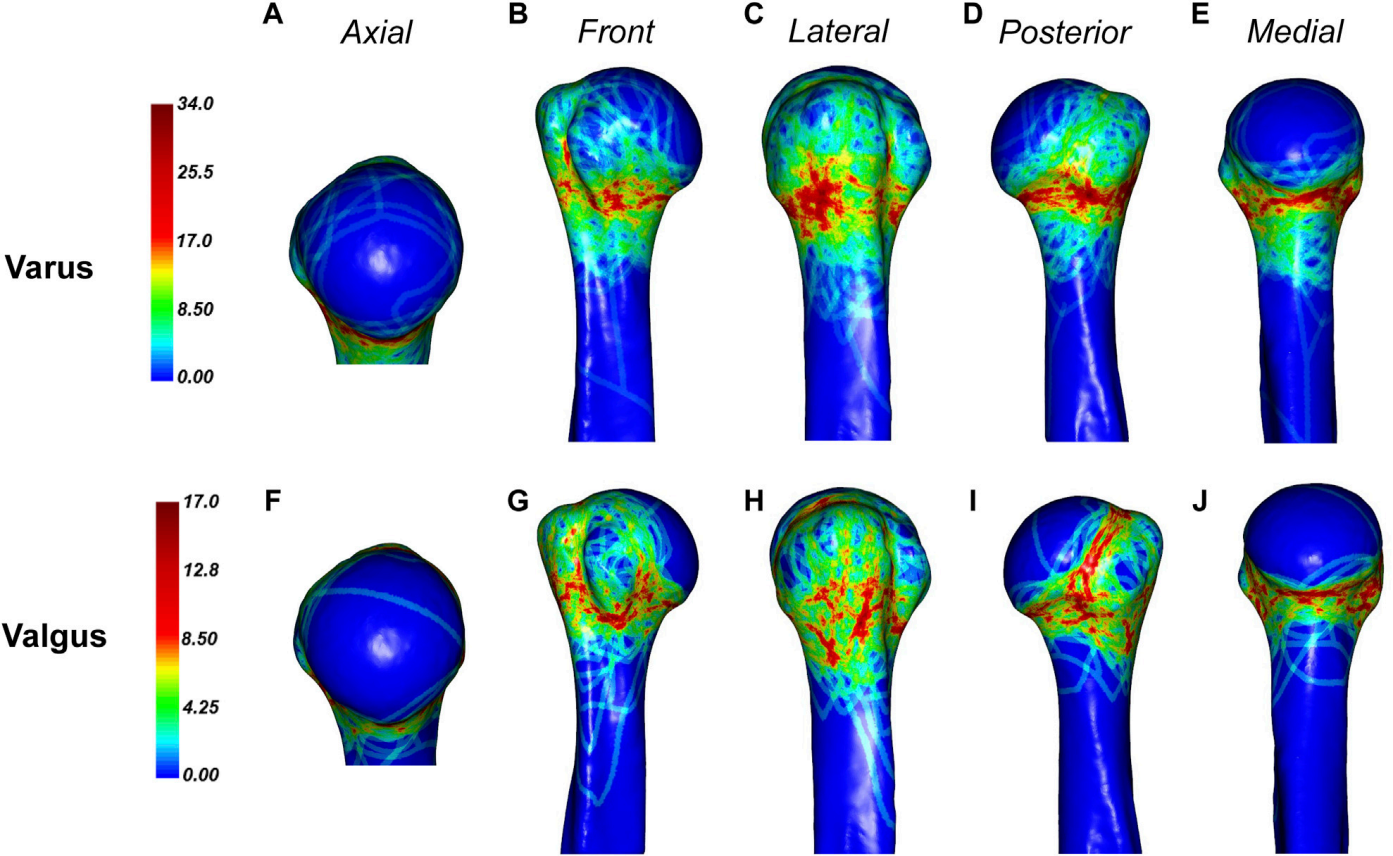
General maps of the hot zones of 3D fracture lines of varus and valgus PHFs. **(A–E)**, 3D heat mapping superimposed with varus PHFs lines (*n* = 88). **(F–J)**, 3D heat mapping superimposed with valgus PHFs lines (*n* = 39), including the axial, front, lateral, posterior, and medial views. Red represents a higher frequency of fracture line density.

The distribution of fractures differs slightly between varus and valgus fractures (Figure 9). In the varus fracture, the fracture is mainly distributed along the surgical neck, less frequently involving the anatomical neck, and is more sparsely distributed anteriorly below the greater tuberosity and below the lesser tuberosity. The medial humeral neck with varus fracture is often combined with fracture insertion. In contrast, the fracture line of the valgus fracture is predominantly concentrated in the lower edge of the lesser tuberosity and the lower, posterior, and upper parts of the anatomical neck, and the greater tuberosity contains some scattered irregular fracture dense zones.

Varus fractures are more common because most patients fall forward when they are injured, which results in the palms or elbows changing from abduction to adductive landing and the body tilting to the affected side, with the shoulders of the affected side landing with it. At this time, the distal end of the fracture is adducted, while the proximal humerus is abducted and externally rotated. In addition, the pull of rotator cuff and deltoid muscle means varus angular deformity is formed. Valgus fractures are caused by the impact of fragments of the humeral head on the lateral or posterolateral side of the metaphysis. When a valgus fracture occurs, the greater tuberosity, lesser tuberosity, or metaphysis is often embedded into humeral head. At this time, restoration of insertion can restore the normal head angle. Understanding these characteristics will contribute to the reduction of fractures (Resch et al., 2016).

The reason for the different fracture line morphology in different age groups may be that PHFs in younger patients is usually caused by high-energy injuries, such as car accidents, while older patients are usually caused by low-energy injuries, such as falls (Baker et al., 2022) Fractures caused by high-energy injuries are more comminuted and therefore more likely to involve the anatomical neck and bicipital groove.

Improved understanding of the morphology of PHFs is critical for preoperative planning and selection of fixation strategies. The fracture rarely encroaches on the humeral head, which has a high bone density and provides a strong anchorage point for the screw (Saitoh et al., 1994). Kamer et al. (2016) analyzed the distribution of bone density within the humeral head and found that it decreased rapidly with increasing distance from the cortical surface. A biomechanical study by Fletcher et al. (2019) predicted the risk of screw perforation by simulating different screw lengths and discovered that the further the screw tip was from the joint, the greater the risk of cutting, especially in osteoporotic patients. These investigations demonstrate that longer screws provide a stronger fixation. It is generally accepted that the distance between the tip of the screw and the articular surface should be 5–8 mm (Ciric et al., 2019). Using a longer screw within this range can prevent the screw from loosening without increasing the risk of screw penetration.

The medial and postmedial aspect of the proximal humerus are dense areas of fracture lines. The medial and posteromedial metaphyseal extensions that are still linked to the humeral head are needed for residual perfusion of the head (Hertel et al., 2004). Therefore, the reduction of fragments on the medial and posteromedial sides of the humeral head is very important. In addition, maintaining medial column support of the proximal humerus can effectively reduce the incidence of postoperative complications of PHFs (Jung et al., 2015; Sears et al., 2020). When there are medial bone defects or comminuted fractures that prevent medial cortical support, the combined use of bone grafting, bone cement augmentation, and posterior or medial auxiliary plates can improve the mechanical stability of the locking plate and reduce postoperative complications (Schliemann et al., 2015; Kim et al., 2018). In addition, intramedullary nail in the treatment of PHFs, intraoperative blood loss, operation time, fracture healing time is better than locking plate (Shi et al., 2019). There are also some new implants for treating PHFs. A clinical study by Fidanza et al. found that using low-profile plate with enhanced fixation properties to treat PHFs can reduce the incidence of postoperative complications (Fidanza et al., 2022b).

It is worth noting that virtual, augmented, 3D printed and mixed reality technologies are widely used in both trauma and arthroplasty (Fidanza et al., 2022a). Virtual surgical and 3D printing can be used to perform virtual reduction of the fractured before surgery, and the fracture fragments can be 3D printed which allows the surgeon to confirm the details of the fracture, determine the morphology of the fracture, the number and location of fragments, and the potential presence of bone defects (Chen et al., 2018; Cocco et al., 2019; Fidanza et al., 2022a). Additionally, appropriate implants were determined on the basis of simulated surgery. These procedures facilitate restore the fragments accurately, shorten surgical time, can reduce X-ray exposure to patients and doctors, allow for selection of appropriate implants during surgery, and optimize surgical outcomes (Marongiu et al., 2020; Fidanza et al., 2022a). Clinical studies by Fidanza et al. and Chen et al. have shown that in the treatment of PHFs, the use of computer-assisted virtual surgery technology and 3D printing technology is more convenient and efficient, can optimize surgical results, shorten operation time, and have higher patient compliance. and showed better results in terms of preoperative planning (Chen et al., 2018; Fidanza et al., 2024).

Knowledge of the morphological features of fractures can facilitate the design of fracture models in biomechanical research. The PHF model is usually performed by osteotomy at the surgical neck to simulate a two-part fracture of the surgical neck, and then the stability between the humeral head and the humeral shaft is tested when fixed only by plate or intramedullary nail (Jabran et al., 2018). However, the position of the osteotomy has not yet been agreed. In this study, the region with the highest fracture line weight is clarified; thus, the results of this study can be used as reference for laboratory modeling in future biomechanical and finite element analysis. According to the fracture heat map, we suggest that the osteotomy gap should be immediately below the lower edge of the articular surface by approximately 5–10 mm (Figure 4). Biomechanical studies based on our proposed fracture model may draw different conclusions compared with previous studies.

The novel classification emphasizes the amounts of fragments contiguous to the humeral head, which is important information regarding the risk of damage to the vascular system of the humeral head. The less soft tissue attachment to the humeral head, the greater the probability of humeral head necrosis (Papakonstantinou et al., 2016; Wang et al., 2021). In addition, the novel classification describes the inclination of the humeral head relative to the humeral shaft, which not only reflects the biomechanical mechanism of the fracture, but also provides an indication of the degree of periosteal damage and displacement, and this is important for blood supply and fracture reduction (Resch et al., 2016). The value of the angle requiring surgical treatment is still under debate. It is reported that a deformity of more than 20° is related to the loss of postoperative reduction and significant damage to clinical results (Solberg et al., 2009; Jung et al., 2015; Jabran et al., 2019).

Classification may be complex, but allows for a more comprehensive definition of fracture without negatively affecting the reliability and reproducibility of the classification process (Marongiu et al., 2020). Regarding interobserver reliability in our novel classification system, an overall kappa value of 0.639 (95% CI, 0.568–0.710) was achieved. In this study, the head tilt analysis showed good interobserver reliability (k > 0.8) and a similar conclusion was reached in the study by Resch et al. (k > 0.85). Therefore, there was no significant decrease in reliability with the classification system of this study, even with the increase in categories. However, reliability was slightly lower for large nodules in this study despite the availability of CT scans (k = 0.704).

This study does have some limitations. First, this is a retrospective study, we did not record the medical history and prognostic data of each patient in detail; Second, for various reasons, some patients are not included in the study, which may bias our results; Third, the process of reconstructing the fracture model and mapping in the software inevitably contains inaccuracies. The guiding significance of fracture classification for the treatment and prediction of prognosis of PHFs needs further study. A larger sample of prospective studies is needed to verify the accuracy of the fracture map in this study and the effectiveness of this new classification system.

## Conclusion

In this study, the fracture line and morphological characteristics of PHFs were clarified in detail by 3D mapping technique. This provides a basis for optimizing internal fixation design, developing a new classification, and standardizing the fracture model of PHFs. By analyzing the morphological characteristics of 220 PHFs, we propose a novel classification system for PHFs that may help doctors assess the degree of fracture damage. In addition, we propose a standardized fracture model for PHFs.

## Data Availability

The original contributions presented in the study are included in the article/Supplementary Material, further inquiries can be directed to the corresponding author.
